# Fli1 and Tissue Fibrosis in Various Diseases

**DOI:** 10.3390/ijms24031881

**Published:** 2023-01-18

**Authors:** Elena V. Mikhailova, Irina V. Romanova, Alexei Y. Bagrov, Natalia I. Agalakova

**Affiliations:** 1 Sechenov Institute of Evolutionary Physiology and Biochemistry, Russian Academy of Sciences, 44 Thorez Avenue, 194223 Saint-Petersburg, Russia; 2 Padakonn Pharma, 20309 Narva, Estonia

**Keywords:** Fli1, fibrosis, angiogenesis, systemic sclerosis, uremic cardiomyopathy, preeclampsia, Ewing’s sarcoma

## Abstract

Being initially described as a factor of virally-induced leukemias, Fli1 (Friend leukemia integration 1) has attracted considerable interest lately due to its role in both healthy physiology and a variety of pathological conditions. Over the past few years, Fli1 has been found to be one of the crucial regulators of normal hematopoiesis, vasculogenesis, and immune response. However, abnormal expression of Fli1 due to genetic predisposition, epigenetic reprogramming (modifications), or environmental factors is associated with a few diseases of different etiology. Fli1 hyperexpression leads to malignant transformation of cells and progression of cancers such as Ewing’s sarcoma. Deficiency in Fli1 is implicated in the development of systemic sclerosis and hypertensive disorders, which are often accompanied by pronounced fibrosis in different organs. This review summarizes the initial findings and the most recent advances in defining the role of Fli1 in diseases of different origin with emphasis on its pro-fibrotic potential.

## 1. Introduction

Fli1 (Friend leukemia integration 1), a member of the ETS transcription factors family, was first described as a factor implicated in the etiology of virally-induced leukemia, Friend murine leukemia virus-induced erythroleukemia [1,2]. Since then, Fli1 has been shown to be highly expressed in immune cells, fibroblasts, endothelial cells, and various cells around blood vessels and it has been recognized to exert a crucial role in a diverse array of normal biological functions including cell proliferation, growth, differentiation, migration, and apoptosis. Accordingly, Fli1 has been reported to be one of the key factors regulating tissues development and regeneration, proper organ growth, and homeostasis. In the immune system, Fli1 is involved in the functioning of immune cells by controlling their development, proliferation, activation, and migration, as well as by regulating cytokines and chemokines [3]. In hematopoietic tissues, Fli1 is one of the key regulators of proliferation of stem/progenitor cells and their differentiation into mature blood cells of different lineages [4]. In the vascular system, Fli1 controls the survival of endothelial cells and vascular development by transcriptional modulation of the expression of angiogenesis-related genes [5,6]. A great contribution to understanding the role of Fli1 for all of these vitally important functions came from investigations on genetically modified mice [7,8]. The crucial role of Fli1 in the regulation of the genes necessary for vascular remodeling and vessel maturation was confirmed by the fact that Fli1 knockout mice die during embryogenesis due to impaired hematopoiesis and the loss of vascular integrity leading to cerebral hemorrhage [7]. 

During angiogenesis, new vessels are formed from existing ones by budding the capillary endothelium. Fli1, being the most highly expressed transcriptional factor in endothelial cells alongside with Erg and GATA-2, regulates this process by controlling the expression of key angiogenesis factors. The expression of Fli1, as well as of Erg and ELF-1, stimulates endoglin [9]. Activated endoglin provides the responses of cells to a signal from TGF-β. Fli1/Erg, when interacting with GATA2, is also indirectly involved in the regulation of the vascular endothelial-statin/epidermal growth factor-like 7 (VE-statin/egfl7) gene [10], which is strongly and specifically expressed in embryonic and mature endothelial cells and is responsible for the proliferation of pericytes and smooth muscle cells, two key points in the stabilization of newly formed capillaries [11]. In endothelial cells, Fli1 is directly regulated by ETS-1, with both ETS genes determining the fate of cells [12].

**Figure 1:**
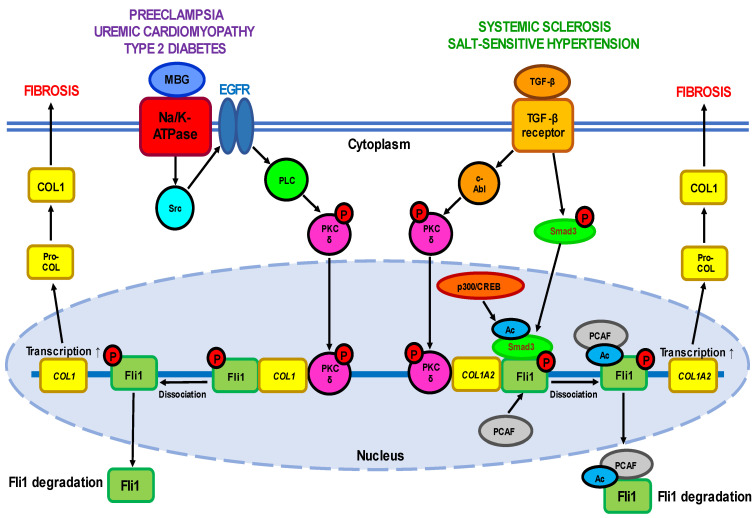
A simplified scheme of the Fli1-dependent pro-fibrotic pathways described for different diseases. One of the pathways is induced by interaction of active TGF-β with its plasma membrane receptor and depends on the ratio between the *COL1*-suppressive activity of Fli1 and the stimulatory ability of Smad3. TGF-β-dependent activation leads to phosphorylation of c-Abl which in turn induces nuclear translocation of PKC-δ. In the nucleus, PKC-δ phosphorylates Fli1 at threonine 312 thus facilitating the acetylation of Fli1 at lysine 380 by PCAF (p300/CREB-binding protein-associated factor). These events result in dissociation of Fli1 from promoter of the *COL1A2* gene and its rapid proteolysis, followed by increased transcription of the *COL1* gene. On the other hand, activation of TGF-β receptor phosphorylates Smad3 which moves into the nucleus and undergoes acetylation by the p300/CREB-binding protein which increases its DNA binding capacity with the promoter of the *COL1A2* gene. The second pathway is triggered by binding of MBG to the plasma membrane Na-K-ATPase, which results in phosphorylation of tyrosine kinase Src followed by sequential phosphorylation/stimulation of PLC and PKC-δ. Active PKC-δ is translocated to the nucleus where it disrupts Fli1 repression of the *COL1* gene leading to the loss of Fli1 activity and excessive collagen synthesis.

However, dysregulated expression of Fli1 due to genetic mutations was recognized as one of the causative factors leading to malignant transformation of cells and the progression of multiple hematological and solid cancers [13], including Ewing’s sarcoma [14] and prostate cancer [15]. Moreover, over the past decades Fli1 has attracted great attention for its role in the etiology of several autoimmune diseases. Sustained Fli1 downregulation is associated with the development of systemic sclerosis, while overexpression of the *FLI1* gene is a well-acknowledged event in systemic lupus erythematosus [3]. Abnormal Fli1 activity is implicated in the development of hypertensive disorders such as uremic cardiomyopathy [16] and preeclampsia [17]. Next, Fli1 deficiency was identified as an essential component of the signaling pathways underlying pro-fibrotic processes, a common phenomenon accompanying the progression of many diseases. Experiments using genetically engineered rodents, in vitro *FLI1* gene silencing, dominant interference, and DNA binding mutants have indicated that Fli1 inhibition is mediated by both direct (DNA binding) and indirect (protein–protein interaction) mechanisms [8,12,18]. Over the past few years, a new trend in investigations increasingly reports the genetic predisposition and epigenetic modifications of genes related to Fli1-dependent pathologies. Besides genetics, epigenetics has been suggested as playing a pivotal role in the development and clinical manifestations of diseases [19,20,21]. Aberrant expression of Fli1 might be responsible for disturbed functioning of a series of genes including that encoding integrins, growth factor receptors, chemokines, and adhesion molecules. This review provides a comprehensive summary of the discovery and current understanding of the role of Fli1 in the progression of diseases of different nature with a particular focus on recent advances in its function associated with pro-fibrotic processes (Figure 1).

## 2. Diseases Associated with Fli1 Deficiency

### 2.1. Fli1 in Systemic Sclerosis

*Fli1 as inductor of fibrosis.* Systemic sclerosis (SSc or scleroderma) is a complex and highly heterogeneous autoimmune disease starting from dysregulation of the immune system and inflammation and followed by impaired angiogenesis, widespread vascular injury, and blood coagulation defects [22]. Persistently activated interstitial fibroblasts induce irreversible fibrosis of the skin and multiple internal organs. Except skin lesions, SSc is accompanied by cardiac defects (lesions in the coronary arteries, pericardium, and myocardium, and myocardial fibrosis) and renal failure (vascular lesions and fibrosis in the kidney, damage to renal glomeruli, and impaired glomerular filtration).

Although the etiology of this life-threatening disorder is not completely understood, SSc-associated vasculopathy was demonstrated to result from hyperactivation of vascular endothelial cells due to genetic or environmental factors leading to dysregulated vascular remodeling. The central mechanism underlying tissue fibrosis is excessive deposition of extracellular matrix (ECM) proteins and impaired collagen homeostasis. Activated interstitial fibroblasts and immune cells synthesize excessive amounts of collagens (first of all types I, III, VI, and VII) and other ECM components such as fibronectin, tenascin-C (TNC), and alpha smooth muscle actin (αSMA), profibrotic cytokines including TGF-β and connective tissue growth factor CTGF (also known as CCN2), accompanied by reduced expression of ECM-degrading enzymes MMP (matrix metalloproteinases) 1 and 3 [23,24].

The causative relationship between transcription factors from the ETS family and pro-fibrotic processes was first established in the pioneering work of Czuwara-Ladykowska et al. [25], which demonstrated that Fli1 plays a crucial role in the regulation of collagen synthesis in dermal fibroblasts by competing with Ets-1, and the ratio of Fli1 to Ets-1 in the presence of co-regulatory proteins may control collagen production. Further in vitro studies have confirmed that *FLI1* suppression induces SSc-like phenotypes in dermal fibroblasts, dermal dendritic cells, keratinocytes, endothelial cells, and macrophages, while in vivo findings have established that Fli1 deficiency is an important mediator of SSc-associated fibrosis [26,27,28]. The role of Fli1 as a natural negative inhibitor of collagen genes was supported by the fact that Fli1 protein levels were inversely proportional with COL1A1 mRNA and collagen 1 levels in the dermal fibroblasts of patients with SSc and in cultured fibroblast isolated from Fli1−/−, Fli1+/−, and Fli1+/+ mice embryos [29]. In the lesional and non-lesional skin of SSc patients, Fli1-positive fibroblasts are either consistently absent or only occasionally seen, and Fli1 immunoreactivity is significantly reduced in endothelial cells [29]. Fli1 is downregulated in dermal dendritic cells [30] and in the primary skin fibroblasts of SSc patients [31]. Furthermore, Fli1 has been demonstrated to play an important role in the regulation of other ECM proteins’ synthesis, including MMP-1 and CTGF [32,33]. Fli1 deficiency impairs the expression of various genes both in fibroblasts and endothelial cells and may represent a unique pathogenetic link between dermal fibrosis and peripheral vasculopathy in SSc. The critical role of Fli1 in the development of this disease was confirmed in the works on *FLI1*-mutated animals such as Fli1+/− mice [8] or that with a conditional deletion of *FLI1* in endothelial cells (Fli1 CKO mice) [34].

*Fli1-DNA binding.* Under normal physiological conditions, Fli1 and Ets-1 form DNA–protein complexes with the sequences of the *COL1A2* gene promoter. The suppressive interaction of Fli1with *COL1* gene is functionally enhanced by co-regulating factors such as Sp1 and Sp3 [25]. Stable *FLI1* transfection leads to dramatic suppression of COL1A2 mRNA and newly synthesized collagenous proteins by inhibiting *COL1A2* promoter activity, while Ets-1 activates the *COL1A2* promoter, which indicates that Fli1 inhibits the *COL1A2* gene by displacing Ets-1. Recent studies have suggested that Fli1 also binds to promoters of genes encoding other proteins such as CCN2 [32], pro-angiogenic adipokine chemerin involved in inflammation, angiogenesis and energy metabolism [35], and one of the damage-associated molecular patterns (DAMPs) molecules S100A12 [36]. 

*Fli1 signaling pathways*. The transcriptional potential of Fli1 is strictly regulated by a balance between the activities of two cytokines—pro-fibrotic TGF-β, a well-established inductor of ECM proteins’ synthesis, and anti-fibrotic TNFα which prevents uncontrolled ECM production. TGF-β stimulation leads to activation of a few cellular targets (Figure 1, Table 1). Active PKC-δ is translocated to the nucleus and recruited to the *COL1A2* promoter, where it directly phosphorylates Fli1 at threonine 312 [37]. Phosphorylation at this residue is necessary for interaction of Fli1 with p300/CREB-binding protein-associated factor (PCAF) resulting in acetylation of Fli1 at lysine 380 [38,39]. Acetylated Fli1 dissociates from the *COL1A2* promoter and degrades, while decreased Fli1 transcription enhances the expression of *COL* genes. Another downstream effector of TGF-β is nonreceptor kinase c-Abl which activates the nuclear translocation of PKC-δ thus facilitating Fli1 acetylation and decreasing Fli1 repression of the *COL1A2* gene [40]. Indeed, persistent stimulation of the c-Abl/PKC-δ/Fli1 cascade at least partially implicates in the fibrotic program in dermal fibroblasts obtained from patients with both SSc and its relative disease—localized scleroderma (LSc) [41]. Moreover, the c-Abl–PKC-δ–Fli1 pathway is activated by TGF-β-dependent stimulation of endothelin-1 [42].

A variety of other factors contribute to SSc due to aberrant expression of Fli1 (Table 1). Among them are cathepsins, proteolytic enzymes regulating angiogenesis and ECM degradation. Enhanced expression of endothelial cathepsin B and cathepsin L due to Fli1 deficiency has been shown to be associated with SSc vasculopathy, whereas downregulation of CTSB, CTSL, and CTSV in dermal fibroblasts implicates in SSc dermal fibrosis [43,44,45]. Next, Fli1 acts as a repressor of CTGF growth factor, an important regulator of angiogenesis, chondrogenesis, and wound healing. Downregulation of Fli1 upregulated the expression of CTGF mRNA which mimics to some extent the profibrotic effects of transforming growth factor TGF-β such as the upregulation of the *COL1A1* and *COL1A2* genes and the downregulation of the *MMP1* gene [32]. Fli1 is a strong inhibitor of a pleiotropic growth factor progranulin (PGRN), an endogenous antagonist of tumor necrosis factor (TNF) receptors, and Fli1 suppression was shown to be associated with upregulated expression of PGRN and TNF-α in the skin of dsSSc patients, Fli1+/− mice, and bleomycin (BLM)-treated mice [46,47].

Besides, Fli1 was shown to regulate the activity of a few angiogenesis-related chemokines with pro- or anti-angiogenic properties in the context of SSc-associated vasculopathy. For instance, Fli1 deficiency is associated with suppression of CXCL5 in the dermal blood vessels of SSc patients, Fli1 siRNA transfected endothelial cells, and Fli1 knockout mice [48]. In contrast, the progression of vasculopathy and fibrosis in SSc heart, lung, and skin due to Fli1 deficiency in fibroblasts and endothelial cells was accompanied by upregulation of CXCL6 [49], while CXCL13 (a chemokine for B cells, follicular T cells, T helper 17 cells, and regulatory T cells) expression was enhanced by Fli1 deficiency in peritoneal murine macrophages [50]. CXCL4, a chemokine with anti-angiogenic capacity, may contribute to peripheral SSc vasculopathy by downregulating Fli1 via c-Abl signaling in endothelial cells. Furthermore, CXCL4 blocked the cell proliferation of HUVECs induced by TGF-β or platelet-derived growth factor (PDGF) [51]. Using three experimental models, namely skin biopsy samples of SSc patients, cultured human dermal microvascular endothelial cells (HDMECs), and a BLM-induced mice SSc model of skin fibrosis, Ikawa et al. [52] showed that the development of SSc vasculopathy is associated with increased expression of endothelial CCR6 due to Fli1 deficiency in dermal small vessels irrespective of disease subtypes and disease duration, while CCL20 expression was significantly elevated in dermal fibroblasts of patients with early dcSSc. 

Fli1 has also been demonstrated to be implicated in the regulation of activities of a wide variety of genes involved in different aspects of cell physiology. Fli1 deletion altered the expression of the proteins involved in vascular homeostasis including VE-cadherin, platelet endothelial cell adhesion molecule 1 (PECAM-1), MMP-9, platelet-derived growth factor B (PDGFB), and S1P(1) receptor in human, Fli1+/− mice, and Fli1 CKO mice dermal microvascular endothelial cells [34]. Fli1 deficiency was suggested to contribute to the suppression of RALDH1 (aldehyde dehydrogenase 1 family member A1) in the dermal dendritic cells (DCs) of patients with diffuse cutaneous SSc and BLM-treated Fli1+/− mice [30]. Another Fli1 target is pro-angiogenic adipokine chemerin, which is involved in inflammation, adipogenesis, angiogenesis, and energy metabolism. The silencing of Fli1, which binds to the chemerin promoter, induced chemerin expression in HDMECs, while Fli1+/− mice exhibited elevated chemerin expression in dermal blood vessels [35]. Fli1 deficiency was shown to be linked with elevated expression of calcium-binding protein S100A12 at the protein level in the epidermis of SSc-involved skin and at the mRNA level in the bulk skin [36]. Hypercoagulation conditions associated with tissue fibrosis and impaired peripheral circulation in SSc was shown to result from Fli1-dependent downregulation of endothelial protein C receptor (EPCR) playing a critical role in the regulation of coagulation system and mediating various cytoprotective effects [53]. The development of SSc-associated pulmonary vascular hypertension was suggested to be linked with Fli1-deficiency-induced upregulation of adipsin, serine proteinase catalyzing the breakdown of complement factor C3 [54].

*Genetic and epigenetic modulations.* During the last few years, SSc development has been shown to greatly depend on the complex interaction between genetic predisposition which determines the susceptibility to SSc and epigenetic modifications which modulate the severity of disease, especially pro-fibrotic processes. *FLI1* is genetically and epigenetically suppressed in SSc patients, suggesting Fli1 deficiency as an important factor in SSc etiology [19,20,21,55]. Fli1+/− mice exhibit an enhanced deposition of type I collagen in the skin and develop mild vascular lesions, while BLM-treated Fli1+/− mice develop skin fibrosis and vascular pathologies [8]. However, the exact mutations associated with SSc have virtually not been explored. The only human genetic variant found to influence Fli1 expression is the polymorphism of (GA)n (GA dinucleotide repeat sequence) microsatellites located at 5′ UTR of the *FLI1* gene [56]. Patients possessing (GA)n alleles with ≥ 22 repeats exhibit decreased Fli1 mRNA levels in the peripheral blood but greater susceptibility to both diffuse cutaneous and limited cutaneous SSc in comparison to healthy controls with (GA)n alleles ≤21.

Growing evidence has suggested that not only inheritance but also epigenetic modifications can mediate the emergence and persistence of SSc phenotypes (Table 1). Fli1 was shown to be a target of all known epigenetic mechanisms, from DNA methylation and histone acetylation to non-coding RNA (microRNA and long non-coding RNA). Thus, compared with the controls, skin biopsy specimens and fibroblasts of SSc patients exhibited hypermethylation of CpG islands and reduced acetylation of histones H3 and H4 in the promoter region of the *FLI1* gene, as well as significantly higher levels of DNA methyltransferase DNMT1, histone deacetylases HDAC1 and HDAC6, proteins MBD-1 and MBD-2 (proteins containing a methyl-CpG DNA binding domain), and MeCP2 (protein of the methylated CpG binding domain) [57]. Genome-wide DNA methylation analysis of skin fibroblast from patients with diffuse and limited cutaneous SSc demonstrated a substantial number of hypomethylated genes relative to ECM proteins’ turnover, including a series of collagen genes (*COL23A1*, *COL4A2*, *COL8A1*, *COL16A1*, *COL29A1*, *COL1A1*, *COL6A3,* and *COL12A1*), *PAX9* encoding the pro-α2 chain of type I collagen, *ITGA9* encoding α integrin, *TNXB* encoding tenascin-XB, *ADAM12* encoding metallopeptidase, and transcription factors gene *RUNX1-3* [58].

Except chromatin remodeling, a variety of miRNAs regulating cell grow differentiation and immune functions have been shown to be implicated in SSc-dependent fibrotic processes. Knockdown of pro-fibrotic miR-26a, which was predicted to target the *FLI1* untranslated region, increased Fli1 expression and decreased collagen I and fibronectin expression in SSc skin fibroblasts, while miR-26 transfection reduced the Fli1 level and enhanced collagen I and fibronectin accumulation [31]. The synthesis of collagen has also been shown to be regulated by a series of miRNAs with pro- or anti-fibrotic properties. Among the pro-fibrotic ones is miR-21, whose expression is upregulated in both dcSSc- and TGF-β-treated fibroblasts [59]. Anti-fibrotic miR-29a, known to bind the 3′ UTR of the *COL1A1* gene, modulates collagen production in an opposing way. Its level was significantly decreased in SSc fibroblasts, SSc skin biopsies, and SSc skin samples from BLM-treated mice, while its overexpression in SSc fibroblasts suppressed collagen synthesis [59,60]. Other anti-fibrotic miRNAs, miR-196a and miR-let-7a, were found to be downregulated both in dermal fibroblasts and the skin of SSc patients thus resulting in abnormal collagen expression [61,62]. In addition, collagen production in SSc fibroblasts was enhanced by the suppression of miR-135b, while the serum and monocytes of SSc patients exhibited diminished levels of miR-135b due to methylation [63].

### 2.2. Fli1 in Uremic Cardiomyopathy

Uremic cardiomyopathy (UC) is a multifactorial pathological condition developed in the patients with chronic kidney disease (CKD) and is responsible for increased rates of heart failure and high mortality, particularly sudden cardiac death [64]. It is important that the risk of developing cardiovascular defects is even higher than that of kidney failure. UC is characterized by remodeling of cardiac tissues leading to hypertrophy of left ventricle and diastolic dysfunction, and by a myriad of metabolic maladaptations in myocardial cells including disturbed mitochondrial function, changes in substrate utilization and metabolic transporter function, and altered insulin response.

The attempts to understand the cardiac perturbations accompanying CKD have revealed its link with abnormal Fli1 expression and increased collagen production as well (Figure 1). Early work has demonstrated that Fli1 transgenic mice developed a high incidence of immunological renal disease and ultimately died of renal failure caused by tubulointerstitial nephritis and immune-complex glomerulonephritis [65]. Later studies have strongly implicated the transcription factor Fli1 in the progressive cardiac fibrosis seen with experimental uremia. The works on rats that underwent partial nephrectomy have shown that many of the clinical features of experimental UC are accompanied by an increase in circulating concentrations of marinobufagenin (MBG), one of the cardiotonic steroids [66,67]. Moreover, MBG signaling through Na/K-ATPase is directly responsible for suppression of Fli1 and the stimulation of cardiac fibroblasts to produce increased amounts of collagen, thus triggering cardiac fibrosis seen with experimental renal failure [66,67]. These findings were confirmed by the fact that intraperitoneal administration of monoclonal antibodies to MBG restored Fli1 expression and reduced cardiac fibrosis [68]. Isolated cardiac fibroblasts cultured with 1 nM MBG in vitro increased procollagen-1 expression accompanied by synthesis of procollagen-1 mRNA and collagen translation [67]. The stimulation of fibroblasts with MBG was prevented by treatment with inhibitors of tyrosine phosphorylation, Src activation, EGFR transactivation, and N-acetyl cysteine. In general, the signaling pathway leading from MBG to increased collagen synthesis resembles that observed in other Fli1-deficiency-associated diseases such as SSc including translocation of PKC-δ to the nucleus where it stimulates Fli1 [16].

### 2.3. Fli1 in Preeclampsia

Another example of pathology at least partially associated with aberrantly low Fli1 expression and the development of vascular fibrosis is preeclampsia (PE), a hypertensive complication of pregnancy adversely affecting both mothers and newborns and in some cases leading to maternal stroke and death [17,69,70]. PE is believed to depend on pre-existing cardiovascular abnormalities and is associated with a life-long risk of hypertension and coronary artery disease [70]. Among others, SARS-CoV-2 infection has recently been suggested to be linked with higher PE incidence, most probably due to inflammatory responses which can infect the placenta and impair its normal development [71]. An important role in PE belongs to maternal and placental vascular dysfunction, in particular abnormal remodeling of spiral arteries, endothelial damage, and an imbalance between the release of pro- and antiangiogenic factors (such as vascular endothelial growth factor A VEGFA, placental growth factor PlGF, soluble fms-like tyrosine kinase 1 sFLT-1, endoglin ENG, and soluble sENG) [72]. 

On the other hand, the progression of PE is closely related to enhanced synthesis of MBG and the development of cardiovascular fibrosis involving Fli1-dependent mechanisms [17,69]. Clinical studies have demonstrated that in umbilical arteries and placentas obtained from PE patients, the expression of Fli1 was significantly (4–9-fold) diminished, while the levels of pro-collagen and collagen-1 increased (2.5–3-fold) [73,74,75]. Similar results were obtained in the work on pregnant Sprague Dawley rats in which PE was induced experimentally by exposure to water with excessive NaCl amounts (1.8 %). The development of a PE-like phenotype in animals was accompanied by a seven-fold decrease in Fli1 expression and a four-fold increase in collagen-1 synthesis in thoracic aortas [76]. Finally, silencing of the *FLI1* gene by 24 h incubation of the isolated segment of healthy human umbilical arteries with Fli1 siRNA not only suppressed the expression of Fli1 at the protein level, but also led to pronounced elevation of pro-collagen-1 and collagen-1 levels [77].

A causative relationship between MBG and Fli1 was confirmed in experiments in vitro on explants of umbilical arteries from healthy women. Incubation of the vascular fragments with 1-10 nM MBG for 24–48 h resulted in a considerable (4-5-fold) decrease in Fli1 levels and accumulation (2–3-fold) of pro-collagen-1 and collagen-1, thus mimicking the processes typical for PE and linked with vascular fibrosis [73,74,75]. In contrast, the degree of collagen-1 accumulation was diminished after the pre-treatment of arterial rings from normotensive pregnant women with monoclonal anti-MBG antibodies [74]. Application of canrenone, an active metabolite of spironolactone used in hypertensive therapy, fully restored Fli1 expression and partially suppressed collagen-1 synthesis in umbilical arteries from PE patients and in the vessels of healthy women incubated with MBG [75]. Moreover, short-term administration of anti-MBG antibodies to rats with experimental PE reversed Fli1 inhibition, although it had no effect on collagen-1 synthesis [76]. However, the underlying molecular pathways of PE in human or rat vessels were not associated with the stimulation of TGF-β and Smad proteins (Figure 1), although the protein level of cytoplasmic PKC-δ increased.

### 2.4. Fli1 as a Common Causative Factor of Hypertension

Interestingly, the patients with preeclampsia [17], chronic renal failure [68,78], and type 2 diabetes [79] shared the same phenotype features, namely the development of cardiovascular fibrosis due to Fli1 deficiency. We assume that the etiology and underlying molecular mechanisms of all these disorders are common and at least in part include enhanced levels of circulating MBG and inhibition of membrane enzyme Na/K-ATPase. The phenomena of MBG-induced fibrosis are sensitive to pharmacological modulation of Na/K-ATPase activity with anti-hypertensive agents [75]. The binding of MBG to Na/K-ATPase initiates the signal transduction that complexes with Src and epidermal growth factor receptor (EGFR) that leads to translocation of PKC-δ to the nucleus and Fli1 repression [16]. However, this process does not involve TGF-β—Smad proteins cascade, although TGF-β antagonist SB-431542 suppressed the stimulation of collagen production [67]. In contrast, Dahl salt-sensitive rats [80] and normotensive salt-loaded Sprague Dawley rats [81] react to a high-salt diet with fibrotic changes in myocardial and renal tissue which are accompanied by the activation of TGF-β [81]. Although the association of MBG and TGF-β described in these experimental models is clear, the causative relationship between them in salt sensitivity has not been explored in details. Interestingly, the crosstalk between Fli1 and TGF-β profibrotic signaling via PKC-δ has previously been described, for example, in type 2 diabetes during salt loading [79]. The development of diabetes is associated with profound inhibition of Fli1 in vascular tissues, while exposure of these rats to high salt levels activates TGF-β and suppresses the expression of Fli1 [79].

**Table 1:** Summary of Fli1-associated pro-fibrotic effects.

	Expression	Effect	Experimental System	References
***Fli1 repressors***				
TGF-β	↑	Activation of TGF-β receptors	Human dermal fibroblasts; rat diabetes model; rat hypertension model	[25,29,32,38,39,79,80,81]
c-Abl	↑	PKC-δ phosphorylation	Human normal and SSc dermal fibroblasts	[40,41,47,51]
PKC-δ	↑	Fli1 phosphorylation	Human normal and SSc dermal fibroblasts; *FLI1*-transfected HEK293T cells; *FLI1* silencing in human UA	[37,39,42,47,77]
Endothelin-1	↑	Fli1 activation	Human normal and SSc dermal fibroblasts; BLM-treated mice	[42]
PCAF	↑	Fli1 acetylation	Human dermal fibroblasts; *FLI1*-transfected HEK293T cells	[38]
CXCL4	↑	Vasculopathy	HUVECs	[51]
MBG	↑	PKC-δ activation; Fli1 suppression	Wild-type and *FLI1*-knockdown mice; rat CKD model; rat cardiac fibroblasts; human cardiac fibroblasts; human renal fibroblasts; *FLI1*-transfected renal fibroblasts; rat diabetes model; rat hypertension model; rat PE model	[16,66,67,68,73,74,75,76,77,79,80,81]
***Fli1-deficiency targets***				
*COL1*	↑	Collagen synthesis	*FLI1*-transfected human dermal fibroblasts; human normal and SSc dermal fibroblasts; murine Fli11−/−, Fli1+/− and Fli1+/+ fibroblasts; wild-type and *FLI1*-knockdown mice; rat CKD model; rat cardiac fibroblasts; human cardiac fibroblasts; human renal fibroblasts; *FLI1*-transfected renal fibroblasts; rat diabetes model; rat hypertension model; rat PE model; human PE UA; *FLI1* silencing in human UA; human MBG-treated UA	[8,16,25,29,32,38,66,67,68,73,74,75,76,77,79,80,81]
CTGF(CCN2)	↑	*COL1A1* and *COL1A2* upregulation; *MMP-1* downregulation	*FLI1*-transfected human dermal fibroblasts; human SSc fibroblasts	[32,33]
MMP-1	↓	Increased production of EMC components	*FLI1*-transfected human dermal fibroblasts; human SSc fibroblasts	[32,33]
Fibronectin	↑	Fibrosis	Human CD14^+^ monocytes and CD14^+^ macrophages	[23]
Chemerin	↑	Impaired angiogenesis	Human SSc dermal vessels and HDMECs; murine Fli1+/− dermal blood vessels;	[35]
S100A12	↑	Skin sclerosis	Human SSc skin;	[36]
RALDH1	↓	Fibrosis	Human dermal dendritic cells; BLM-treated Fli1+/− mice	[30]
Cathepsin B	↑ ↓	Vasculopathy Increased production of EMC components	*FLI1* silencing in HDMECs; Fli1+/− mice; human dcSSc dermal fibroblasts, early dcSSc dermal fibroblasts	[43]
Cathepsin L	↑ ↓	Vasculopathy Increased production of EMC components	*FLI1* silencing in HDMECs; human SS dermal vessels and skin; *FLI1*-knockout mice; skin of BLM-treated mice	[45]
Cathepsin V	↓	Increased production of EMC components	*FLI1* silencing in HDMECs; human dcSSc dermal fibroblasts, microvascular ECs, dcSSc and lcSSc skin keratinocytes	[44]
PGRN	↑	Inflammation; skin sclerosis; fibrosis	Human SSc dermal fibroblasts; Fli-1+/− mice; BLM-treated mice; human LSc skin lesions	[46,47]
TNF-α	↑	Inflammation; fibrosis	Human LSc skin lesions;	[47]
CXCL5	↓	Vasculopathy	Human dcSSc dermal blood vessels; *FLI1* silencing in HDMECs; dermal vessels of *Fli1* knockout mice	[48]
CXCL6	↑	Tissues fibrosis; vasculopathy	Human SSc dermal fibroblasts; *FLI1* silencing in HDMECs	[49]
CCL20	↑	Fibrosis	Human early dcSSc	[52]
CCL6	↑	Vasculopathy	Human SSc dermal vessels; *FLI1* silencing in HDMECs	[52]
EPCR	↓	Impaired vascular homeostasis	Human SSc dermal vessels; Fli1+/− mice; *FLI1* silencing in HDMECs	[53]
VE-cadherin	↓	Impaired vascular homeostasis	MDMECs of Fli1 CKO mice; MDMECs of Fli1+/− mice; *FLI1* silencing in HDMECs	[34]
PECAM-1	↓	Impaired vascular homeostasis	MDMECs of Fli1 CKO mice; MDMECs of Fli1+/− mice; *FLI1* silencing in HDMECs	[34]
MMP-9	↓	Impaired vascular homeostasis	MDMECs of Fli1 CKO mice; MDMECs of Fli1+/− mice; *FLI1* silencing in HDMECs	[34]
PDGF-B	↓	Impaired vascular homeostasis	MDMECs of Fli1 CKO mice; MDMECs of Fli1+/− mice; *FLI1* silencing in HDMECs	[34]
S1P1	↓	Impaired vascular homeostasis	MDMECs of Fli1 CKO mice; MDMECs of Fli1+/− mice; *FLI1* silencing in HDMECs	[34]
Adipsin	↑	Vascular hypertension	Human SSc dermal vessels; *FLI1* silencing in HDMECs	[54]
***Genetic and epigenetic factors***				
(GA)n alleles	↑	Increased susceptibility to SSc	Human SSc peripheral blood	[56]
Acetylation of histones H3 and H4 in the *FLI1* gene promoter	↓	*FLI1* suppression; increased collagen synthesis	Human skin, normal and dcSSc fibroblasts	[57]
HDAC-1 and 6	↑	*FLI1* suppression	Human skin, normal and dcSSc fibroblasts	[57]
MBD-1 and 2, MeCP2	↑	DNA methylation	Human skin, normal and dcSSc fibroblasts	[57]
Methylation of CpG islands in the *FLI1* promoter	↑	*FLI1* suppression; increased Collagen synthesis	Human skin, normal and dcSSc fibroblasts	[57]
DNMT1	↑	*FLI1* suppression	Human skin, normal and dcSSc fibroblasts	[33,57]
*COL23A1, COL4A2* methylation	↓	Increased collagen synthesis	Human dcSSc and lcSSc dermal fibroblasts	[58]
*ITGA9* methylation	↓	TGF-β upregulation	Human dcSSc and lcSSc dermal fibroblasts	[58]
*ADAM12* methylation	↓	TGF-β upregulation	Human dcSSc and lcSSc dermal fibroblasts	[58]
miRNA-26a	↑	*FLI1* suppression	Primary SSc skin fibroblasts	[31]
miRNA-21	↑	TGF-β upregulation; collagen synthesis	Human dcSSc and TGF-β treated normal fibroblasts	[59]
miRNA-29a	↓	TGF-β upregulation; collagen synthesis	Human dcSSc and TGF-β treated normal fibroblasts	[59]

Abbreviations. dcSSc—diffuse cutaneous systemic sclerosis; lcSSc—limited cutaneous systemic sclerosis; LSc—localized scleroderma; CKD—chronic kidney disease; BLM—bleomycin; HDMECs—human dermal microvascular endothelial cells; HUVECs—human umbilical vein endothelial cells; MBG—marinobufagenin; PE—preeclampsia; UA—umbilical artery; CTGF (connective tissue growth factor also known as CCN2); PGRN—progranulin; EPCR—endothelial protein C receptor; DNMT1—DNA methyltransferase; MBD-1 and 2—proteins containing methyl-CpG DNA binding domain, MeCP2—protein of the methylated CpG binding domain; HDAC-1 and 6—histone deacetylases; *ITGA9*—integrin 9 gene.

## 3. Fli1 Overexpression as a Cause of Ewing’s Sarcoma

Ewing’s sarcoma (ES), belonging to the family of Ewing’s sarcoma family of tumors (ESFT), is a highly aggressive and very metastatic tumor arising predominantly in the bones and soft tissues of children and young adults [82,83]. The major mechanism driving ESFT development is a genetic *FLI1*-dependent process, but it is different from that underlying Fli1-deficiency-associated disorders. It involves a generation of oncogenic fusion protein EWS–FLI1 functioning as an aberrant transcriptional factor that dysregulates the activity of several target genes thus leading to initiation of malignant cell transformation and tumor growth [84,85]. Although the progression and onset of ES is generally not considered to be linked with fibrosis, recent studies have demonstrated an impaired regulation of ECM metabolism providing the microenvironment for growing tumors [86,87]. 

*Oncogenic Fli1-dependent mechanisms*. In the vast majority of cases, the ESFT is associated with reciprocal chromosomal translocation which fuses the N-terminal transcriptional activation domain of the Ewing’s sarcoma breakpoint region 1 (*EWSR1*) gene located on chromosome 22 with the C-terminal DNA binding domain of the friend leukemia virus integration 1 (*FLI1*) gene on chromosome 11—t(11;22) (q24;q12) [88,89]. The result of this mutation is the generation of chimeric oncogenic factor EWSR1–FLI1 with two primary domains, EWS domain being a potent translational regulator and the FLI1 domain containing a highly conserved DNA binding domain [90]. The EWS–FLI1 fusion protein possesses phase transition properties that allow it to transition into a liquid phase and interact with non-membrane organelles. A series of in vivo and in vitro studies has shown that specific EWS–FLI1 binding domains recognizes and preferentially bind to highly repetitive GGAA-containing microsatellite sequences near targeted genes to activate or repress them [91,92]. At least three consecutive GGAA motifs are necessary for EWS–FLI1 interaction and at least two distinct classes of GGAA microsatellites, “enhancer-like” and “promoter-like”, have been identified. Upon binding to otherwise non-functional GGAA microsatellite, EWS–FLI1 transforms inactive silent regions of chromatin into an accessible state, creates de novo enhancers, and transcriptionally activates oncogenic gene expression [93]. EWS/FLI1’s responsiveness to oncogenesis appears to depend on microsatellite length [91,92].

*Fli1 and ECM proteins in ES*. The reorganization of the ECM network associated with tumorigenesis and the progression of ES has been linked with dysregulation of various extracellular proteins, glycoproteins, and signaling molecules. Watanabe et al. [86] found that three of six ES cell lines and four of six primary tumors exhibited high expression levels of tenascin-C (TNC). In vivo assays have established that excessive TNC synthesis is the result of direct interaction of the EWS–FLI1 fusion protein with four ETS binding sites at the *TNC* promoter. Moreover, the levels of endogenous TNC mRNA and protein decreased following inhibition of EWS–FLI1 fusion protein expression [86]. Recently, the causative link between EWS–FLI1 oncogenic activity and the regulatory role of TNC in the growth of the extracellular matrix was confirmed by the work of He et al. [94]. 

A few other targets of EWS/FLI1 are involved in the disruption of ECM’s structural integrity. The work [95] has shown that the malignant transformation of NIH-3T3 cells by the EWSR1-FLI1 oncoprotein requires the enhanced activity of ERK1/ERK2 protein kinases. Recently, this ERK upregulation was linked to dysregulated catabolism of heparan sulfate proteoglycan, one of the cell surface molecules playing a crucial role in the regulation of cell differentiation, adhesion, and migration [96]. Another EWS–FLI1 effector is caveolin-1 (CAV1) which is able to induce metastasis in ES via the pathway including MEK/ERK cascade and matrix metalloproteinase-9 (MMP-9) [96]. Lysyl oxidase (LOX), an enzyme implicated in the proper maintenance of the structural integrity of the extracellular matrix, is not expressed in ES cells and primary tumors due to downregulation by the EWS/FLI1 oncoprotein [97]. Interestingly, a small population of ES cells undergoing phenotypic switch to a more metastatic state exhibited enhanced Wnt/β-catenin activity, which antagonizes EWS–FLI1-dependent inhibition of the TGF-βII receptor leading to increased susceptibility of cells to TGF-β ligands [98]. 

*Fli1-dependent epigenetic modulations.* Functioning as a potent oncoprotein, EWS–FLI1 not only binds to chromatin but also alters its organization, thus changing the expression of a large number of genes necessary for ES development using various epigenetic mechanisms [93,99]. These processes are carried out by enzymes affecting both DNA methylation (mainly at the sites interacting with transcription factors) and histone acetylation (resulting in unfolding of DNA which is normally tightly wrapped around histones) which makes DNA more accessible for transcription factors and, accordingly, enhances the transcription of genes in this region. The result of GGAA microsatellite binding is the recruitment of acetyltransferase p300 regulating chromatin state by acetylating H3K27 at its binding sites, and MLL (mixed-lineage leukemia) complexes that methylate H3K4 [99]. These processes loosen DNA packaging and facilitate access to the genes to initiate transcription. Later work has confirmed that aberrant expression of the EWS–FLI1 oncogene was shown to be accompanied by selective deposition of histone markers H3K4me3, H3K9ac, and H3K27ac at its promoter region [100]. One of the critical EWS–FLI1 target gene required for ES proliferation is *NR0B1* (nuclear receptor subfamily 0 group B member 1) containing a GGAA-microsatellite-enriched region near the transcriptional start site and functioning as a transcriptional regulator [92]. The examples of other EWS–FLI1 oncogenic effectors are various regulatory genes encoding proteins acting at the G1 stage of the cell cycle such as cyclin G1, cyclin D1, p21, and p27 [101]. Metastatic ES spread was shown to be associated with EWS/FLI1 binding to the promoter of histone methyltransferase EZH2 (Enhancer of Zeste (Drosophila) Homolog 2) which regulates stemness genes such as the nerve growth factor receptor (NGFR) or genes involved in neuroectodermal and endothelial differentiation (*EMP1*, *EPHB2*, *GFAP*, and *GAP43*) [102]. In a human stem cell models of tumor initiation, EWS–FLI1 was suggested to increase the expression of *HOX* (homeobox) genes crucial for correct embryonic development, and overexpression of a few *HOX* genes downstream of EWS–FLI1 has been linked to loss of H3K27me3 and the gain of H3K4me3 markers at the promoters of *HOX* genes [103].

Moreover, the role of EWS–FLI1 in ES is not limited to transcriptional regulation, but also includes modulation of the expression of microRNAs (miRNAs) and long non-coding RNAs by direct inhibition. Thus, EWS–FLI1 represses the promoter activity of miRNA-145 which functions as an activator of RNA-induced silencing complexes (RISCs). Such inhibition leads to an increase in cell pluripotency, reduces their differentiation, and enhances carcinogenesis [104]. Another target of the EWS–FLI1 protein is the transcriptional suppressor miRNA-22, whose expression participates in the inhibition of cell proliferation programs. This leads to overexpression of the H3K9me1/2 histone demethylase KDM3A (JMJD1A/JHDM2A) and tumor growth [105]. Other direct targets of EWS–FLI1 which are constitutively repressed in ES are miRNA-214-3p and let-7a [106,107]. All of the Fli1-associated oncogenic effects are summarized in Table 2.

## 4. Perspectives

Analyzing the literature data collected to date, it becomes clears that aberrant expression of Fli1 is an important trigger of various life-threatening disorders. It makes this transcriptional factor an attractive target for molecular therapy. However, currently there are no therapeutic or gene engineering strategies able to directly restore the expression level of Fli1 in the cells of patients with Fli1-dependent diseases. For example, the traditional treatment of Ewing’s sarcoma is not specific and includes DNA damaging chemotherapeutic drugs such as doxorubicin and cyclophosphamide or radiation therapy (reviewed by Daley et al. [108]). Since the EWS–FLI1 fusion protein is a difficult target for therapeutic approaches due to its lack of a peculiar binding pocket for chemical agents or enzymatic activity, the search for ES treatment has been focused on alternative strategies such as inhibition of critical FLI1-interacting enzymes. One of proposed methods is the inhibition of the DNA damage response protein and transcriptional coactivator PARP1 (Poly (ADP-ribose) polymerase) whose expression was shown to be directly driven by the EWS–FLI1 fusion product [109]. Disrupting EWS–FLI1:PARP1’s interaction with the PARP1 inhibitor olaparib suppressed the growth of ES cells, primary mouse tumor xenografts, and tumor metastases, while tumorigenesis in an EWS–FLI1-induced ES mouse xenograft model was completely inhibited by combination of olaparib and the second-line chemotherapeutic drug temozolomide. Another potential target in ES is USP9X (ubiquitin-specific peptidase 9 X-linked) which binds to the ETS domain of EWS–FLI1 and stabilizes its protein expression (Wang, 2023) [110]. Treatment of ES cells with USP9X inhibitor WP1130 induced de-ubiquitination and depletion of EWS–FLI1 in vitro and in vivo accompanied by the reduced growth of ES cells and tumors. Erkizan et al. [111] reported a small molecule, YK-4-279, which was able to induce apoptosis and suppress the growth of ES orthotopic xenografts by blocking interaction between EWSR1–FLI1 and RNA helicase A (RHA). The first molecule believed to be a direct inhibitor of EWSR1–FLI1 is TK216, a YK-4-279 derivative, which recently entered phase II clinical trials in ES patients either as monotherapy or in combination with vincristine [112]. Recently, the gene engineering approach has led to the construction of a lentivirus that can specifically transduce ES cells and sensitize them to otherwise relatively non-toxic (Val)ganciclovir by increasing the expression of viral thymidine kinase, thus inducing a strong anti-tumorigenic effect [113]. 

With regard to systemic sclerosis, the therapeutic approaches targeting Fli1 are even more limited. To date, a few non-specific drugs have been tested. Thus, glycyrrhizin used for the treatment of hepatic diseases and itching dermatitis was shown to possess an anti-fibrotic effect in SSc dermal and lung fibroblasts via downregulation of Dnmt1, upregulation of Fli1, and induction of MMP1 gene expression via an ERK1/2-dependent mechanism [114]. Another compound is fluoroquinolone antibiotic ciprofloxacin which was able to decrease the levels of DNA methyltransferase 1 (Dnmt1) and increase the expression of Fli1 in dermal fibroblasts obtained from SSc patients [33]. Cyclophosphamide was demonstrated to exert a beneficial effect on Fli1 deficiency-dependent vasculopathy in a murine SSc model by improving the expression of endothelial Fli1, as well as angiogenesis and vasculogenesis effectors [115]. Recently, the traditional Chinese medicine Bushen Yijing decoction (BSYJ) was proposed for SSc treatment because of its ability to exert an anti-fibrotic effect in a mouse SSc model and primary human SSc fibroblasts by suppressing miRNA-26a expression which restores Fli1 levels [31]. However, the effectiveness of these drugs is still controversial. Therefore, rational therapeutic targeting of Fli1 represents a major challenge. Further advances in the knowledge of Fli1’s key regulators and large prospective studies are urgently needed to provide a basis for the development of new treatment approaches.

## Figures and Tables

**Table 2:** Fli1-associated oncogenic effects.

	Expression	Effect	Experimental System	References
Tenascin-C (TNC)	↑	ECM growth	*FLI1* transfection of human fibrosarcoma HT-1080 cells; human primary ES tumors; *TNC*-knockout cell lines; murine xenografts	[86,87,94]
ERK1/2 protein kinases	↑	Tumor growth; metastases; dysregulation of EMC metabolism	NIH-3T3 cells; human ES cell lines	[87,95]
Caveolin-1 (CAV1)	↑	Tumor growth; metastases;	Human ES cell lines; *CAV1* silencing; murine xenografts	[96]
MMP-9	↑	Tumor growth; metastases; dysregulation of EMC metabolism	Human ES cell lines; *CAV1* silencing; murine xenografts	[96]
LOX	↓	Tumorigenesis	Human ES cell lines; murine xenografts	[97]
Wnt/β-catenin/TGF-β/SMAD	↑	Altered synthesis of ECM proteins; promotion of angiogenesis in local TME	Human ES cells; HUVECs; *FLI1* knockdown	[98]
DNA methylation	↓	Tumorigenesis	Human ES cell lines; primary pediatric MSC; *FLI1* knockdown; *in vivo* tumorigenesis	[99]
Histone acetylation, H3K4Me3, H3K9ac, H3K27ac markers at the *FLI1* promoter	↑	*FLI1* transcriptional activation; tumorigenesis	Human ES cell lines; primary pediatric MSC; primary tumor cells; *FLI1* knockdown; *in vivo* tumorigenesis	[99,100]
Acetyltransferase p300	↑	Tumorigenesis	Human ES cell lines; primary pediatric MSC; *FLI1* knockdown; *in vivo* tumorigenesis	[99]
Cyclin G1, Cyclin D1	↑	Tumorigenesis	Human SK-N-MC and PNKT-1 cell lines; murine xenografts; *FLI1* transfected NIH3T3 cell	[101]
NR0B1	↑	Increased ES susceptibility	ES A673 and HEK 293EBNA cell lines; *FLI1* knockdown	[92]
Histone methyltransferase EZH2	↑	Tumor development; metastases growth	ES and neuroblastoma cell lines; murine xenografts; RNA interference; *FLI1* transfection	[102]
HOX genes	↑	Aberrant differentiation; tumorigenesis	ES cell lines; HeLa, neuroblastoma, HEK293FT, NCSC, BM-MSCs; *FLI1* transduction of NCSC	[103]
miRNA-145	↓	Tumorigenesis	hpMSCs; MSC; ES cell lines; mouse xenografts	[104]
miRNA-22	↓	Tumor growth	ES A673 cells; miRNA-22 overexpression; murine xenografts	[105]
miRNA-214-3p	↓	Tumor growth	ES cell lines; human BM-MSCs; human ES primary tumors and metastases; *FLI1* silencing	[106]
miRNA-let-7g	↓	Tumorigenesis	MSCs; ES cell lines; murine xenografts; *FLI1* knockdown	[107]

Abbreviations: MSC—mesenchymal stem cells; BM—bone marrow; NCSC—neural crest stem cells; hpMCS—human pediatric MSC.

## Data Availability

No data was used for the research described in the article.
